# A high-throughput neutralizing assay for antibodies and sera against hepatitis E virus

**DOI:** 10.1038/srep25141

**Published:** 2016-04-28

**Authors:** Wei Cai, Zi-Min Tang, Gui-Ping Wen, Si-Ling Wang, Wen-Fang Ji, Min Yang, Dong Ying, Zi-Zheng Zheng, Ning-Shao Xia

**Affiliations:** 1 State Key Laboratory of Molecular Vaccinology and Molecular Diagnostics, National Institute of Diagnostics and Vaccine Development in Infectious Diseases, School of Life Sciences, Xiamen University, Xiamen, Fujian 361005, PR China; 2 State Key Laboratory of Molecular Vaccinology and Molecular Diagnostics, National Institute of Diagnostics and Vaccine Development in Infectious Diseases, School of Public Health, Xiamen University, Xiamen, Fujian 361005, PR China

## Abstract

Hepatitis E virus (HEV) is the aetiological agent of enterically transmitted hepatitis. The traditional methods for evaluating neutralizing antibody titres against HEV are real-time PCR and the immunofluorescence foci assay (IFA), which are poorly repeatable and operationally complicated, factors that limit their applicability to high-throughput assays. In this study, we developed a novel high-throughput neutralizing assay based on biotin-conjugated p239 (HEV recombinant capsid proteins, a.a. 368–606) and staining with allophycocyanin-conjugated streptavidin (streptavidin APC) to amplify the fluorescence signal. A linear regression analysis indicated that there was a high degree of correlation between IFA and the novel assay. Using this method, we quantitatively evaluated the neutralization of sera from HEV-infected and vaccinated macaques. The anti-HEV IgG level had good concordance with the neutralizing titres of macaque sera. However, the neutralization titres of the sera were also influenced by anti-HEV IgM responses. Further analysis also indicated that, although vaccination with HEV vaccine stimulated higher anti-HEV IgG and neutralization titres than infection with HEV in macaques, the proportions of neutralizing antibodies in the infected macaques’ sera were higher than in the vaccinated macaques with the same anti-HEV IgG levels. Thus, the infection more efficiently stimulated neutralizing antibody responses.

Hepatitis E virus (HEV) is a non-enveloped virus with a worldwide distribution and may cause severe acute hepatitis^1^. Its single-stranded, positive-sense RNA genome consists of three open reading frames (ORFs)^2^, among which ORF2 encodes a 660-amino acid viral capsid^3^. A method for evaluating neutralization is needed to assess an effective immune response against the virus. However, there was previously no easy, high-throughput method for the evaluation of anti-HEV neutralization. Current neutralization tests are based on traditional real-time PCR^4^,^5^,^6^,^7^ or the immunofluorescence foci assay (IFA)^8^,^9^. The neutralization assay based on real-time PCR calculates the quantities of virus by detecting RNA. However, real-time RT-PCR is an unstable method for high-throughput detection. (Supplementary Fig. 1). Additionally, IFA ensures that neutralization post-attachment can be tested because only replicating virus is detected. However, it is time-consuming (taking approximately 7 days) and labor-intensive.

Here, we developed a high-throughput method to quantitatively evaluate the neutralization of anti-HEV monoclonal antibodies (mAbs) and sera based on the fluorescence signal of conjugated p239 (HEV recombinant capsid particle, assembled from a.a. 368–606 of pORF2)^10^ instead of unstable HEV virions^11^. p239 presented the immune-dominant neutralization epitopes as native HEV particles^10^ and could be used as a surrogate to study the HEV neutralization and infection process^12^,^13^. This report presents an ideal alternative method for measuring neutralization capacity of sera that it is easily adapted to high-throughput technology.

## Results

### Construction and characterization of biotin conjugated p239

We first conjugated p239 with fluorescein isothiocyanate (FITC) as previously reported^14^, and the cells that had been incubated with the conjugated p239 were directly assessed using high-throughput flow cytometry (FCM, Beckman Coulter CyAn ADP with a HyperCyt Loader, UNC, USA). However, the FITC signal was not sufficiently strong, which resulted in a FITC-p239 input that was greater than or equal to 16.6 μg/mL (Supplementary Fig. 2a). The high input of FITC-p239 meant that the neutralization results were related to the concentration of the antibodies as well as the p239 input (Supplementary Fig. 2b). A large amount of p239 had to be sufficiently neutralized by adding a quantity of serum, which also caused non-specific blocking.

**Figure 1:**
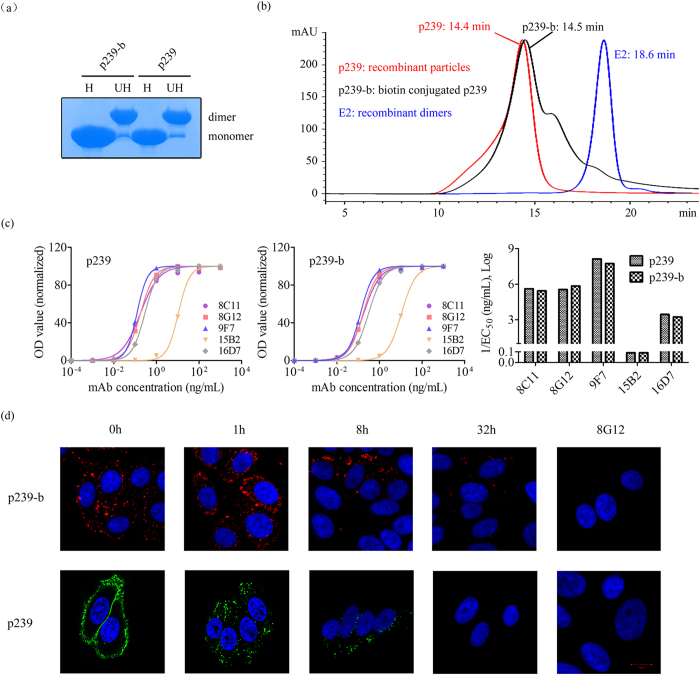
Comparison of p239 and p239-b (biotin-conjugated p239). p239 and p239-b were characterized using SDS-PAGE to analyse dimer formation ((**a**) H: heated samples, UH: unheated samples) via high-performance liquid chromatography (HPLC) using a TSK Gel G5000PWXL 7.8*300 mm column (Tosoh) to analyse particle assembly ((**b**) E2 was used as a non-particle control) and via indirect ELISA to determine their reactivities with five HEV-specific monoclonal antibodies (mAbs): 8C11, 8G12, 9F7, 15B2, and 12A10. The binding affinities were presented as EC_50_ (ng/ml) values. The reciprocal 1/EC_50_ values are shown on the y axis, with larger y values representing higher binding affinities (**c**). The binding and penetration of p239-b and p239 to cells were detected via fluorescence analysis ((**d**), bar is 10 μm).

To improve the detectable signal and to decrease the p239 input, we further conjugated p239 with biotin and used allophycocyanin-conjugated streptavidin (streptavidin APC) (Molecular Probes) to increase the fluorescence signal of p239 in the cells. To determine whether the conjugation influenced the chemical and biological activities of p239, biotin-conjugated p239 (p239-b henceforth) was characterized for dimer presentation, particle assembly, reactivity with anti-HEV mAbs and cell-binding reactivity. Most of the p239-b was present as p239 dimers on sodium dodecyl sulphate polyacrylamide gel electrophoresis (SDS-PAGE) gels (Fig. 1a). Similar retention times were noted for p239-b and p239 via molecular sieve chromatography (Fig. 1b), whereas E2 (a.a. 394–606 of pORF2)^10^, which was present as dimers but not particles, showed a longer retention time. p239-b assembled into particles (Fig. 1b) using dimers as basic units (Fig. 1a), similar to p239. The reactions of p239 and p239-b with five representative mAbs were evaluated by enzyme-linked immunosorbent assay (ELISA). Among these five antibodies, 8C11, 8G12 and 9F7 were neutralizing antibodies that recognized 3 independent conformational antigenic sites on the HEV capsid^6^,^7^,^10^,^12^,^15^. The other two antibodies (15B2 and 12A10) recognized linear epitopes located at a.a. 403–418 and a.a. 423–437, respectively; 12A10 was also demonstrated to be a neutralizing antibody^4^,^12^. Similar reactivity profiles between p239-b and p239 were shown, indicating that the major epitopes on p239-b were not influenced by biotin conjugation (Fig. 1c). Furthermore, the binding and entry process of p239-b on cells was measured and compared with that of p239. HepG2 cells were incubated with p239-b or p239 for 30 min at 4 °C and were then directly harvested or harvested after 1, 8 or 32 h re-culturing at 37 °C. The presence of p239-b was identified with streptavidin APC staining (Fig. 1d, upper panel), and p239 was localized by staining with an anti-HEV mAb and a FITC-conjugated secondary antibody, as previously reported^12^,^13^ (Fig. 1d, lower panel). Here, the virus-like particles were incubated with neutralizing mAb 8G12 as a blocking control (Fig. 1d, right panel). p239 and p239-b attached to the cell surface in significant amounts (Fig. 1d, 0 h) and penetrated the cells from 1 to 8 h after incubation; both p239-b and p239 were degraded at 32 h. These results indicated that biotin conjugation does not affect the particle assembly, antigenicity or the cell interaction activity of p239.

### Optimization of a novel HEV neutralization assay amenable to high-throughput tests

**Figure 2:**
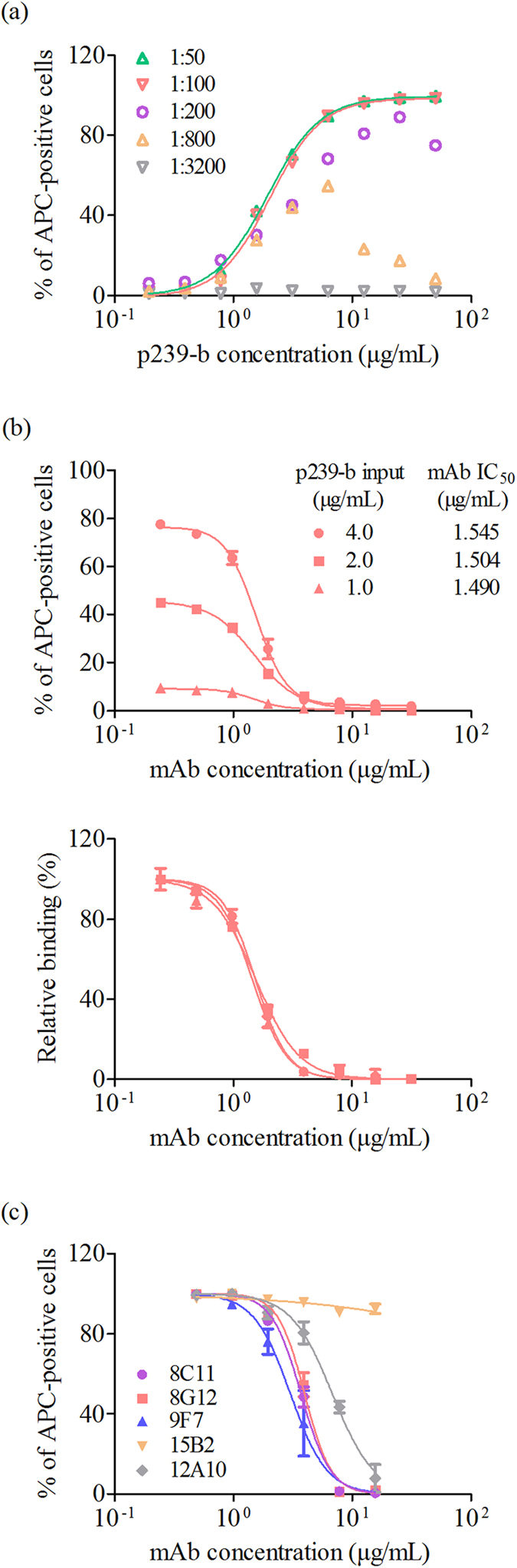
Establishment and feasibility of a neutralization assay based on flow cytometry (FCM). (**a**) The percentages of streptavidin APC-positive cells at different p239-b inputs and different streptavidin APC dilutions are shown. The dose-dependent curves of the p239-b concentration (x-axis) versus the percentage of streptavidin APC-positive cells (y-axis) were slightly under 1:50 and 1:100 dilutions of streptavidin APC, and the EC_50_ of the p239 input was 2.0 μg/mL. (**b**) Evaluation of neutralizing mAb 9F7 with a 1:100 dilution of streptavidin APC at three concentrations of p239-b (upper panel); the percentages of relative binding are shown in the lower panel. The results were consistent at p239-b inputs of 1.0, 2.0, and 4.0 μg/mL. (**c**) Neutralizing capacities of 5 anti-HEV mAbs. The curves were fitted for nonlinear regression (log [inhibitor] vs. normalized response, variable slope).

To confirm the input of p239-b in a neutralization evaluation, we quantitated the binding of p239-b to cells using FCM. Serial 2-fold dilutions of p239-b (starting at 50 μg/mL) were incubated with HepG2 cells in a 96-well culture plate. After washing three times, the cells were re-incubated with different dilutions of streptavidin APC (from 1:50 to 1:3200). As shown in Fig. 2a, when the concentration of streptavidin APC was less than or equal to a 1:200 dilution, the percentage of positive cells was not dependent on the dose of the p239 input, which probably resulted from a lack of sufficient streptavidin APC to stain the p239-b on the cells. The dose-dependent curves were slightly under 1:50 and 1:100 dilutions of streptavidin APC, and the 50% maximal effective concentration (EC_50_) of the p239 input was approximately 2.0 μg/mL under both of these conditions. Therefore, a neutralizing mAb 9F7 was further tested to determine its neutralization titre using this model, with a 1:100 dilution of streptavidin APC and 3 different concentrations of p239 (1.0, 2.0 and 4.0 μg/mL). As shown in Fig. 2b, the 50% inhibitory concentrations (IC_50_ values) of 9F7 were 1.490, 1.504 and 1.545 μg/mL at p239-b concentrations of 1.0, 2.0 and 4.0 μg/mL, respectively. The results followed the “percentage law”, which states that, over a range of virus concentrations, the amount of virus neutralized by a given concentration of antibody is constant as long as the antibody is present in excess over the amount of virus^16^,^17^,^18^. Thus, a p239-b input of 2.0 μg/mL was chosen as a working concentration for the subsequent studies.

### Evaluation of sera samples using the novel assay

The method was then used to evaluate 5 reported anti-HEV antibodies, including neutralizing mAbs 9F7, 8G12, 8C11, and 12A10^4^,^6^,^7^,^12^,^15^ and non-neutralizing mAb 15B2^12^. HEV-specific mAbs were divided into strong (9F7, 8G12, 8C11), weak (12A10) and non-neutralizing (15B2) antibodies (Fig. 2c). The experimental results were consistent with those of previous studies in our laboratory^4^,^6^,^7^,^15^. These results indicated that the assay could be used to assess the neutralizing capacities of antibodies.

**Figure 3:**
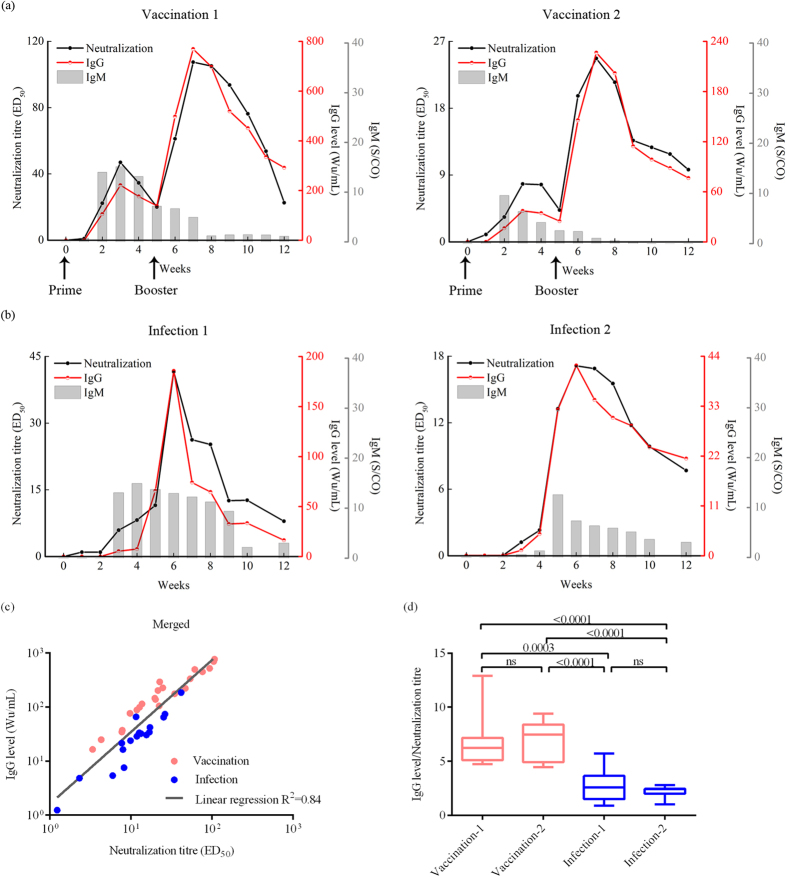
Relationship between neutralizing titres (ED_50_ values) and anti-HEV IgG levels in macaque sera. Neutralization titres and anti-HEV IgG levels in macaque sera after HEV vaccine administration ((**a**) arrows indicate the day when the booster injection was performed) or HEV infection (left panel: HEV genotype 1 virus, right panel: HEV genotype 4 virus) (**b**). Neutralization titres (black line), IgG levels (red line) and IgM S/CO (grey bars) are shown, respectively. (**c**) The neutralization titre was plotted against the corresponding anti-HEV IgG level of macaque sera after either vaccination (n = 11, pink spots) or infection (n = 9, blue spots). Linear regression analysis indicated that there was a high degree of correlation. (**d**) Comparison of the ratio of the IgG level to the neutralization titre (I/N) from serum samples after vaccination or infection using an unpaired t test. The ratio is shown as the range (whiskers), interquartile (boxes), and median (line within the boxes) values. Two-sided P values are given. P < 0.05 was considered significant, and ns indicates non-significant differences.

The method was then used to evaluate the neutralization of serial sera from cynomolgus macaques that were vaccinated with HEV vaccine or infected with HEV. Figure 3a shows neutralization titres, IgG levels and IgM sample/cutoff ratios (S/CO) (Wantai Biopharm, Beijing, China) in two vaccinated macaques. The neutralization titre of the sera substantially increased and reached a first peak 3 weeks after primary inoculation. After one booster dose in week 5, the neutralization titres in the sera increased again and reached a second peak in week 7. The fluctuations in neutralization titres and IgG levels were similar, indicating that IgG represented the major neutralizing antibodies induced by the HEV vaccine. The same phenomenon was observed in HEV-infected macaques, where both the neutralization titres and IgG levels peaked 6 weeks post-infection (Fig. 3b), coinciding with the decline in viral activity, as measured by RNA copies (Supplementary Fig. 3).

However, diversity between neutralization titres and IgG levels was also observed in some weeks; the neutralization titres increased earlier than the IgG levels in the first infected macaque (Fig. 3b, left panel) and decreased more slowly in both infected macaques (Fig. 3b, weeks 7 and 8). Moreover, in both vaccinated macaques, the neutralization titres also increased more dramatically after the primary inoculation (Fig. 3a). A high level of anti-HEV IgM was detected during all stages (Fig. 3a,b), which indicated that IgM also contributed to the neutralization of sera in both vaccinated and infected macaques.

Figure 3c shows the correlation analysis of the samples with both neutralization and IgG-positive status. A strong correlation was again demonstrated between these two markers in both vaccinated (pink spots, n = 11) and infected macaques (blue spots, n = 9). However, it is interesting that the I/N ratios (ratio of IgG level / neutralization titre) of the sera from vaccinated macaques were significantly higher than those from infected macaques; the average of I/N in the vaccinated group was approximately three times higher than the value in the infected group (Fig. 3d). Meanwhile no significant difference in the I/N ratio were observed between two macaques that were treated in the same manner (Fig. 3d). These results suggest that vaccination needs to stimulate a three-fold increase in IgG levels to reach a similar neutralization titre as HEV infection.

### Comparison of the novel and IFA assays

**Figure 4:**
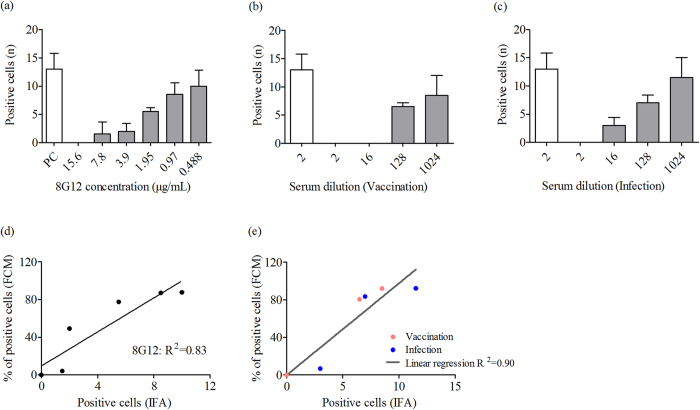
Neutralization tests of 8G12 and macaque sera evaluated by IFA. (**a**) *In vitro* neutralization of Kernow virus by serial two-fold dilutions of 8G12. The open bars represent PBS and the shaded bars represent 8G12. Neutralization assay for serial eight-fold dilutions (beginning with a dilution of 1:2) of sera from cynomolgus macaques vaccinated with the HEV vaccine (**b**) or infected with HEV (**c**). The open bars represent pre-immune sera, the shaded bars represent post-vaccination or post-infection sera, and the whiskers indicate individual results. The reciprocal of the final dilution of serum is given below. The neutralization of 8G12 (n = 6) (**d**) and macaque sera after either vaccination (n = 4, pink spots) or infection (n = 4, blue spots) (**e**) analysed by FCM was plotted against that analysed by IFA. Linear regression analysis indicated that there was a high degree of correlation.

Traditional methods for evaluating HEV neutralization include IFA, which ensures that neutralization post-attachment can be tested because only replicating virus is detected. A comparison between IFA and this novel method was measured with mAbs and sera. The neutralization of 8G12 and macaque sera was verified by IFA (Fig. 4a–c). Figure 4b,c showed that a 1:16 dilution of vaccinated sera collected in week 6 could not detect positive cells, but the same dilution of infected sera could detect positive cells. Thus, the neutralization titres of sera from vaccinated macaques were higher than infected macaques, which was in agreement with the results shown in the left panels of Fig. 3a (ED_50_ = 61.16) and 3b (ED_50_ = 41.57). We verified the relationship between the blocking activity in FCM and the neutralizing activity in IFA using a neutralizing antibody, 8G12 (n = 6) and sera collected from either vaccinated (n = 4, pink spots) or infected (n = 4, blue spots) macaques. A linear regression analysis indicated that there was a high degree of correlation between these two assays (Fig. 4d,e). Therefore, the novel neutralizing assay based on p239 can be used to evaluate the neutralization capacity of sera against hepatitis E virus.

## Discussion

Previous studies have shown that the peptide constructed from a.a. 459–606 of pORF2 (identified as E2s domain, P domain or P2 domain) is the major target of anti-HEV neutralizing antibodies^19^,^20^,^21^. It was also demonstrated that the E2s domain comprised the outer interface of the HEV capsid^21^,^22^,^23^,^24^,^25^ and participated in the virus-cell binding process^12^,^13^. A recombinant particle, p239, displaying this domain could stimulate high titre anti-HEV IgG responses and has successfully been used as the single antigen in a licensed HEV vaccine^26^,^27^. Meanwhile p239 has also been widely used in HEV vaccination and infection studies^4^,^12^,^13^,^14^,^28^. Using the IFA neutralizing assay, we also proved that the major neutralizing antibodies present in sera recognized determinants on p239 (Supplementary Fig. 4). Therefore, in this study, we developed a novel high-throughput neutralizing assay using p239 to simulate the virus and showed a correlation between the novel and IFA assays.

Using this novel method, we quantitatively evaluated the neutralization of sera from HEV-infected and vaccinated macaques. A linear regression analysis indicated that there was a high degree of correlation between the anti-HEV IgG levels and neutralization titres of macaque sera. Further analysis also indicated that the proportions of neutralizing antibodies present in the infected macaques’ sera were higher than those from the vaccinated macaques with the same amount of anti-HEV IgG. This phenomenon may be caused by a difference in epitope exposure between the virions and the vaccine. These results indicate that vaccination should generate more anti-HEV IgG than a natural infection to reach the same neutralization titre. The sera from vaccinated macaques showed substantially higher titres of both IgG and neutralizing antibodies (Fig. 3a–c). This finding explains why the vaccine efficiently protects humans from HEV infection^26^,^27^.

In conclusion, a novel, high-throughput neutralizing assay for HEV was developed in this study. Using this method, we provided direct evidence that neutralization titres of HEV serum samples are associated with anti-HEV IgG levels. However, this study also shows that the neutralization titre and IgG levels are not always correlated. The neutralization titre is also influenced by anti-HEV IgM responses, which further supports the conclusion that this method will be useful in HEV research.

## Materials and Methods

### Ethics statement

The animal experiment was designed based on the principles expressed in the “Guide for the Care and Use of Laboratory Animals” by the National Research Council of the National Academies and “Guidance for Experimental Animal Welfare and Ethical Treatment” by the Ministry of Science and Technology of the People’s Republic of China. The experimental procedures and the animal use and care protocols were carried out in accordance with the guidelines of the Xiamen University Institutional Committee for the Care and Use of Laboratory Animals and were approved by the Committee on Ethical Use of Animals of Xiamen University.

### Cell lines, monoclonal antibodies, hepatitis E vaccine, and hepatitis E virus

HepG2 cells (HB-8065, obtained from the ATCC, MD, USA) were grown in Dulbecco’s modified Eagle’s medium (DMEM) containing 10% fetal calf serum (both from GIBCO, California, USA) and antibiotics (100 units/mL ampicillin and 100 units/mL streptomycin) (Lukang, Shandong, China) at 37 °C with 5% CO_2_. HEV capsid protein-specific mAbs were obtained in our laboratory using a standard murine mAb preparation protocol^15^. Hepatitis E vaccine (Hecolin; Xiamen Innovax Biotech, Xiamen, China) used HEV recombinant capsid protein p239 as the single antigen which was produced in bacterial cells. The HEV viruses were isolated from stool samples from rhesus monkeys infected with HEV genotype 1 virus (strain Xinjiang) and genotype 4 virus (strain Ch-S-1).

### Cloning, expression, purification and labelling of p239

The gene encoding p239 was described previously^19^. Then, the genes were cloned into the pTO-T7 plasmid and transformed into the *E. coli* ER2566 strain (Invitrogen). The transformant was cultured in LB medium at 37 °C for 4 h and then incubated for an additional 4 h in the presence of 0.2 mM isopropylthio-β-D-galactoside (IPTG). The cells were lysed by sonication in the presence of 2% Triton X-100. The sonicate was allowed to stand at 4 °C for 30 min and then centrifuged at 12,000 rpm for 10 min. Then, the precipitant was washed once with 0.2% Triton X-100 and twice with buffer I (200 mM Tris-HCl, pH 8.5; 5 mM EDTA; and 100 mM NaCl). Each wash was followed by centrifugation at 12,000 rpm for 10 min. The pellet was resuspended in 4 M urea buffer (200 mM Tris-HCl, pH 8.5; 5 mM EDTA; 100 mM NaCl; and 4 M urea), allowed to stand for 30 min at room temperature and centrifuged at 12,000 rpm for 10 min. The supernatant was dialyzed against PBS (pH 7.4) overnight and centrifuged at 12,000 rpm for 10 min. p239 was present in the supernatants. FITC and biotin labelling of p239 were performed according to the protocols of Molecular Probes.

### Gel-filtration HPLC

Purified proteins were subjected to chromatography through a TSK Gel G5000PWXL 7.8*300 mm column (Tosoh) equilibrated in PBS, pH 7.45, connected to a 126NM/168NM HPLC system equipped with 508 autosampler (Beckman Instruments) to analyse the molecular mass. The column flow rate was maintained at 0.5 ml/min.

### Indirect enzyme-linked immunosorbent assay (ELISA)

p239 and p239-b were used as coating antigens and were added (100 ng per well) to carbonate buffer (pH 9.6) at 37 °C for 4 h, respectively. After washing once with 0.025% Tween 20 in PBS, the proteins were blocked with ED buffer at 37 °C for 2 h. Serial dilutions of different mAbs were incubated with the protein at 37 °C for 30 min. The plates were washed five times and incubated with HRP-conjugated goat anti-mouse antibodies (Wantai, Beijing, China) at 37 °C for 30 min. After washing five times, the colour was developed, and the reaction was terminated. The absorbance was then measured at 450 and 620 nm.

### Immunofluorescence

HepG2 cells cultured on coverslips were incubated with p239-b or p239 for 30 min at 4 °C, and then directly harvested or harvested after 1, 8 or 32 h re-culturing at 37 °C. The cells were fixed with 4% paraformaldehyde (Sigma Aldrich) and permeabilized with 0.3% Triton X-100 (Amresco) in PBS. The samples were blocked with 10% goat serum in PBS for 1 h, incubated with 15B2 (anti-p239 mouse mAb) for 30 min, and then labelled with fluorescein isothiocyanate-conjugated goat anti-mouse IgG (Molecular Probes) for 30 min, whereas p239-b was directly incubated with streptavidin APC (Molecular Probes). After three washes, the cells were stained with 0.5% 4′,6-diamidino-2-phenylindole (DAPI; Invitrogen). Fluorescence signals were detected with a laser-scanning confocal microscope (LSM 780, Carl Zeiss, Germany).

### Flow cytometry neutralization assay (FCM)

Mixtures of p239-b and serial two-fold dilutions (beginning with a dilution of 1:2) of sera were added to a 96-well culture plate that had been seeded with HepG2 cells (6 × 10^4^ per well) and then incubated at 37 °C for 30 min. After washing three times, the cells were re-incubated with streptavidin APC for an additional 30 min. The cells were then washed with PBS and subjected to FCM (Beckman Coulter CyAn ADP with a HyperCyt Loader, UNC, USA). The mock-adsorbed cells were treated equally and were used for background gating. The percentage of positive cells was measured, and the results were analysed using GraphPad Prism software (GraphPad Software Inc., San Diego CA).

### Immunofluorescence foci assay (IFA)

IFA was performed as previously described^8^,^9^. We seeded 100,000 HepG2/C3A cells per well onto eight-well Lab-Tek II CC2 slides (Nunc) 1 d before infection. Virus was produced from cells that had been transfected with infectious cDNA clones of the HEV strain Kernow (clone P6; GenBank accession no. JQ679013)^29^. Then, 100 μL of the mixture of virus and serial eight-fold dilutions (beginning with a dilution of 1:2) of sera were added to each chamber and incubated for 5 h at 34.5 °C in a CO_2_ incubator. The virus mixture was removed; the cells were washed with PBS; and fresh medium supplemented with 2% DMSO, 100 U penicillin ml^−1^, 0.1 mg streptomycin ml^−1^ and 0.1 mg gentamicin ml^−1^ was added, followed by incubation at 34.5 °C for 5 d. The cells on the eight-well chamber slides were fixed with 4% paraformaldehyde (Sigma Aldrich) and permeabilized with 0.3% Triton X-100 (Amresco) in PBS. The samples were incubated with monoclonal antibody (mAb) 4#, which was donated by Dr Youchun Wang^30^, and labelled with fluorescein isothiocyanate-conjugated goat anti-mouse IgG (Molecular Probes). The stained cells were visualized with a fluorescence microscope and manually counted.

## Additional Information

**How to cite this article**: Cai, W. *et al.* A high-throughput neutralizing assay for antibodies and sera against hepatitis E virus. *Sci. Rep.* **6**, 25141; doi: 10.1038/srep25141 (2016).

## Supplementary Material

Supplementary Information
