# Supplementary material for: A high-throughput neutralizing assay for antibodies and sera against hepatitis E virus
Source: Sci Rep. 2016 Apr 28;6:25141. doi: 10.1038/srep25141 (PMC4848499; doi:10.1038/srep25141)
Supplement: Supplementary Information [file srep25141-s1.pdf]

Supplementary Information

A high-throughput neutralizing assay for antibodies and sera against hepatitis E virus

Wei Cai [1]*, Zi-Min Tang [2]*, Gui-Ping Wen [2], Si-Ling Wang [2], Wen-Fang Ji [1], Min Yang [1], Dong Ying [1], Zi-Zheng Zheng [2] & Ning-Shao Xia [1,2]

[1] State Key Laboratory of Molecular Vaccinology and Molecular Diagnostics, National Institute of Diagnostics and Vaccine Development in Infectious Diseases, School of Life Sciences, Xiamen University, Xiamen, Fujian 361005, PR China

[2] State Key Laboratory of Molecular Vaccinology and Molecular Diagnostics, National Institute of Diagnostics and Vaccine Development in Infectious Diseases, School of Public Health, Xiamen University, Xiamen, Fujian 361005, PR China

* These authors contributed equally to this work

Correspondence and requests for materials should be addressed to Z.Z. (zhengzizheng@xmu.edu.cn) or N.X. (nsxia@xmu.edu.cn)

1. Neutralization assay of antibodies and macaque sera using real-time PCR

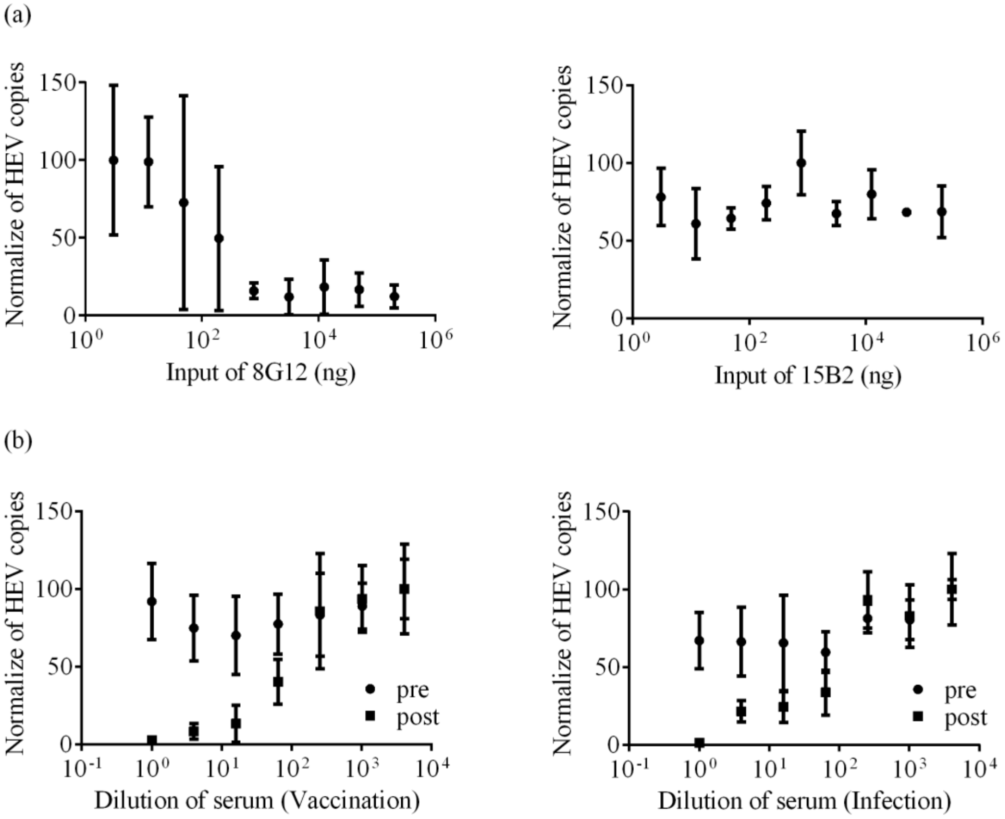

Supplementary Figure 1. Neutralization of antibodies (a, 8G12 and 15B2) and sera from cynomolgus macaques vaccinated with HEV vaccine or infected with HEV (b) was measured using real-time PCR. The antibody input or serum dilutions was plotted against the percentage of viral copies normalized to those in the positive control group.

2. Evaluation of neutralizing mAb 9F7 at three concentrations of FITC-p239

(a)

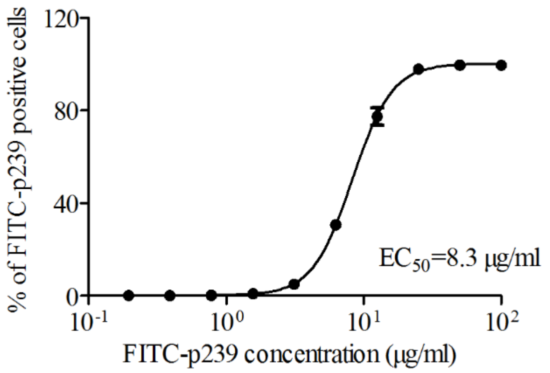

(b)

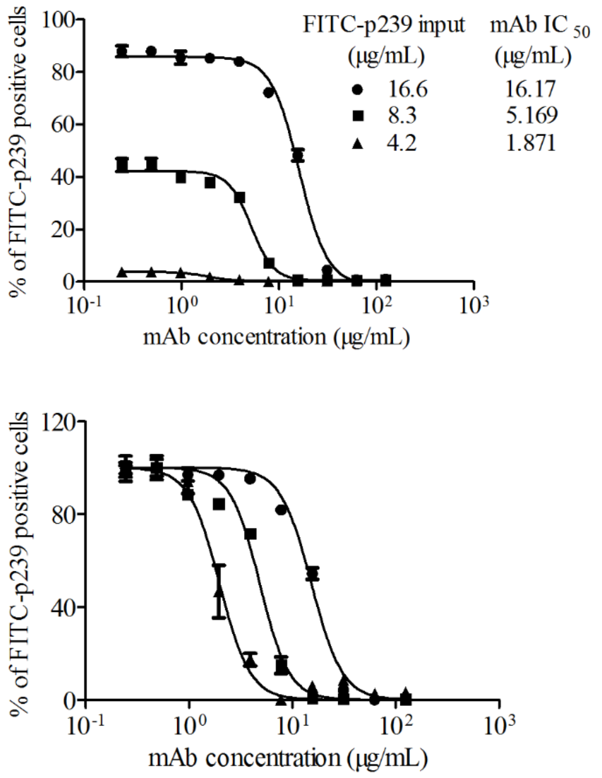

Supplementary Figure 2. (a) Sigmoidal adsorption curves of the FITC-p239 concentration (x-axis) versus the percentage of FITC-positive cells (y-axis) are shown, and the EC_{50} was 8.3 μg/ml. (b) Evaluation of neutralizing mAb 9F7 at three concentrations of FITC-p239 (upper panel); the percentages of relative binding are shown in the lower panel. Non-linear regression analysis of the IC_{50} of mAb 9F7 at

each concentration of FITC-p239 was performed using GraphPad Prism, revealing IC_{50} values of 1.871, 5.169 and 16.17 μg/mL at FITC-p239 inputs of 4.2, 8.3 and 16.6 μg/mL, respectively.

3. Detection of RNA copies and IgG levels in the sera from macaques infected with HEV

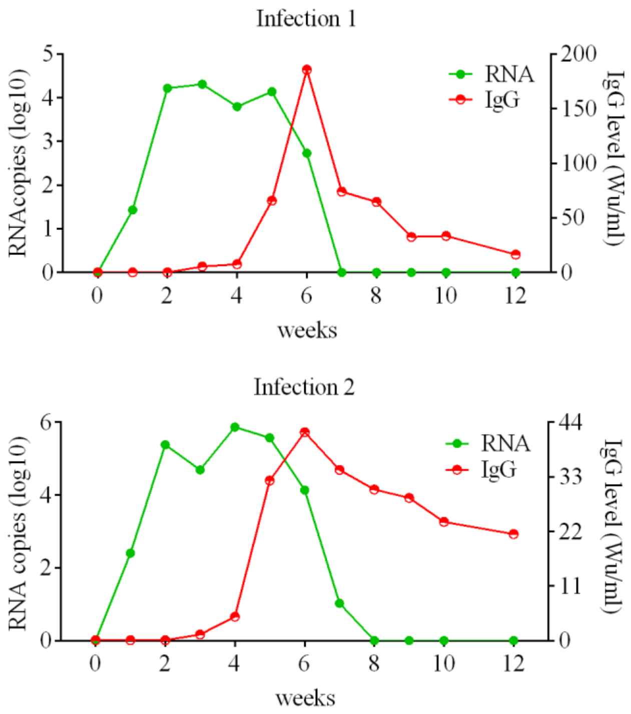

Supplementary Figure 3. HEV RNA copies and anti-HEV IgG levels in macaque sera after HEV infection (upper panel: HEV genotype 1 virus, lower panel: HEV genotype 4 virus). The RNA copies (green line) and IgG levels (red line) are shown.

4. Neutralization assay of sera after incubation with p239 using IFA

Forty micrograms of p239-b was incubated with 400 μg of Dynabeads™ M-280 Streptavidin (Invitrogen) for 30 min at room temperature with gentle rotation. The

mixture was placed in a magnet for 2–3 min, and the supernatant was discarded. The procedures above were repeated and three groups of similarly treated beads were obtained. Then, a 128-fold dilution of vaccinated or infected sera collected in week 6 was successively incubated with three groups of the treated beads at 37 ℃ for 30 min to ensure the complete elimination of antibodies against p239 in the sera. The neutralizing capacities of treated and untreated sera were detected by IFA.

As shown in Supplementary Figure 4, antibodies that recognized the epitopes located on a.a. 368-606 after incubation with p239 were captured by p239, and the post-immune sera could not neutralize HEV, as determined by IFA. This result shows that the antibodies in sera neutralized HEV by blocking HEV binding to cells. Therefore, the novel neutralizing assay based on p239 could be used to evaluate the neutralization capacity of sera against hepatitis E virus.

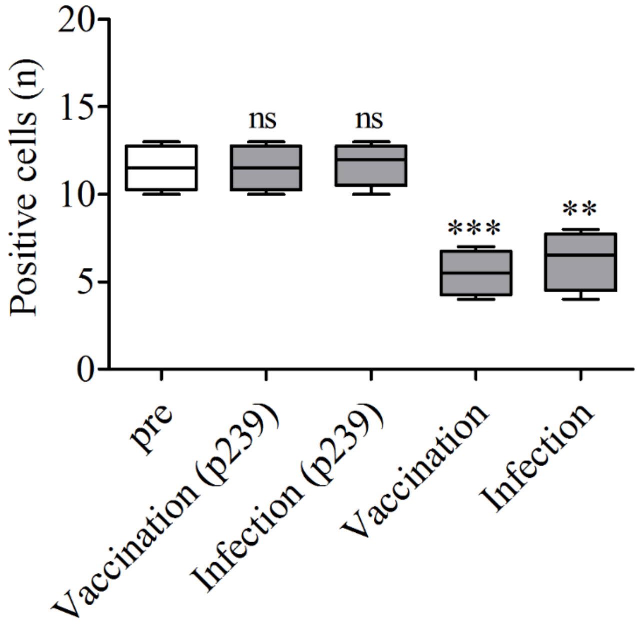

Supplementary Figure 4. Comparison of the neutralization capacity of pre-immune sera (open boxes) with treated and untreated post-immune sera (shaded boxes) from

vaccinated or infected macaques using an unpaired t test. The positive cells are shown as the range (whiskers), interquartile (boxes), and median (line within the boxes) values. Two-sided P values are shown. The asterisks indicate significant differences (***P=0.0006 and **P=0.0027), and ns indicates non-significant differences.
